# Miscellany

**Published:** 1911-01

**Authors:** 


### MISCELLANY

ANATOMIST (MALE).—*Army and Medical Museum,* January 18, 1911.—The United States Civil Service Commission announces an examination on January 18, 1911, at the usual places, to secure eligibles from which to make certification to fill a vacancy in the position of anatomist (male), at $1,600 per annum, in the Army Medical Museum, Office of the Surgeon General, and vacancies requiring similar qualifications as they may occur, unless it shall be decided in the interest of the service to fill the vacancy by reinstatement, transfer, or promotion.

Men only will be admitted to this examination. Age limit, 20 years or over on the date of the examination.

It is desired that the person appointed to this position shall be young, in good health, a graduate in medicine, have a thorough knowledge of pathologic histology, pathology, and bacteriology, be capable of making photomicrographs, understand microscopes, surgical instruments and appliances, and be able to prepare, card, and keep in order museum specimens.

In accordance with a recent act of Congress an applicant for this examination will be required to be examined in the state or territory in which he resides and to show in his application that he has been actually domiciled in such state or territory for at least one year previous to the date of the examination. This examination is open to all citizens of the United States who comply with the requirements.

This announcement contains all information which is communicated to applicants regarding the scope of the examination, the vacancy or vacancies to be filled, and the qualifications required.

Applicants should at once apply either to the United States Civil Service Commission, Washington, D. C., or to the secretary of the board of examiners at the several places where examinations are held, for application and examination Form No. 1312. No application will be accepted unless properly executed, including the medical certificate, and filed with the Commission at Washington. In applying for this examination the exact title as given at the head of this announcement should be used in the application.

As examination papers are shipped direct from the commission to the places of examination, it is necessary that applications be received in ample time to arrange for the examination desired at the place indicated by the applicant. The commission will therefore arrange to examine any applicant whose application is received in time to permit the shipment of the necessary papers.

Issued November 30, 1910.

Physical Director (Male).—The United States Civil Service Commission announces an examination on January 21, 1911, to secure eligibles from which to make certification to fill a vacancy in the position of physical director and assistant disciplinarian at $800 a year at the Haskell Institute, Kansas, and vacancies requiring similar qualifications as they may occur in the Indian Service, unless it shall be decided in the interest of the service to fill the vacancy by reinstatement, transfer, or promotion.

It will not be necessary for competitors to report at any place for examination. Their eligibility for the position will be determined upon the evidence furnished in application and examination Form No. 1312 concerning their education, training and experience. Applications for this position will be accepted only from men who are graduates of medicine.

The duties of the position require ability to organize and direct the work of classes in physical culture in connection with the regular school work, and to assist the disciplinarian in the performance of his duties. Applicants must have reached their twentieth but not their fiftieth birthday on the date of the examination.

Each applicant is required to furnish with his application a medical certificate showing him to be in good health and free from tuberculosis in any and every form. No person will be appointed who is unable to speak the English language. Each applicant must attach to his application a statement showing the number in his family and the number that will require accommodations at the Indian school or agency in case he receives appointment.

This examination is open to all citizens of the United States who comply with the requirements. This announcement contains all information which is communicated to applicants regarding the scope of the examination, the vacancy or vacancies to be filled, and the qualifications required.

Applicants should at once apply to the United States Civil Service Commission, Washington, D. C., for Form 1312. No application will be accepted unless properly executed, including the medical certificate, and filed with the Commission prior to the hour of closing business on January 21, 1911. In applying for this examination the exact title as given at the head of this announcement should be used in the application.

---

Taking Time by the Forelock.—Sourire.

"Tell me the worst, doctor. I can bear it."

"Well, I think I would better bring my bill today. Tomorrow it will be too late."

